# Upgrading the physiochemical and sensory quality of yogurt by incorporating polyphenol-enriched citrus pomaces with antioxidant, antimicrobial, and antitumor activities

**DOI:** 10.3389/fnut.2022.999581

**Published:** 2022-09-26

**Authors:** Soha A. Alamoudi, Ahmed M. Saad, Nouf H. Alsubhi, Ghadeer I. Alrefaei, Diana A. Al-Quwaie, Najat Binothman, Majidah Aljadani, Mona Alharbi, Humidah Alanazi, Ahmad O. Babalghith, Mohammed S. Almuhayawi, Hattan S. Gattan, Mohammed H. Alruhaili, Samy Selim

**Affiliations:** ^1^Biological Sciences Department, College of Science and Arts, King Abdulaziz University, Rabigh, Saudi Arabia; ^2^Biochemistry Department, Faculty of Agriculture, Zagazig University, Zagazig, Egypt; ^3^Department of Biology, College of Science, University of Jeddah, Jeddah, Saudi Arabia; ^4^Department of Chemistry, College of Sciences and Arts, King Abdulaziz University, Rabigh, Saudi Arabia; ^5^Department of Biochemistry, College of Science, King Saud University, Riyadh, Saudi Arabia; ^6^Medical Genetics Department, College of Medicine, Umm Al-Qura University, Makkah, Saudi Arabia; ^7^Department of Medical Microbiology and Parasitology, Faculty of Medicine, King AbdulAziz University, Jeddah, Saudi Arabia; ^8^Yousef Abdullatif Jameel Scientific Chair of Prophetic Medicine Application, Faculty of Medicine, King Abdulaziz University, Jeddah, Saudi Arabia; ^9^Department of Medical Laboratory Sciences, Faculty of Applied Medical Sciences, King Abdulaziz University, Jeddah, Saudi Arabia; ^10^Special Infectious Agents Unit, King Fahad Medical Research Center, King AbdulAziz University, Jeddah, Saudi Arabia; ^11^Department of Clinical Laboratory Sciences, College of Applied Medical Sciences, Jouf University, Sakaka, Saudi Arabia

**Keywords:** citrus, pomaces, antioxidant, antitumor, antimicrobial, yogurt, sensory quality

## Abstract

Industrial pomaces are cheap sources of phenolic compounds and fibers but dumping them in landfills has negative environmental and health consequences. Therefore, valorizing these wastes in the food industry as additives significantly enhances the final product. In this study, the citrus pomaces, orange pomace (OP), mandarin pomace (MP), and lemon pomace (LP) were collected by a juice company and subjected to producing polyphenols and fiber-enriched fractions, which are included in functional yogurt; the pomace powder with different levels (1, 3, and 5%) was homogenized in cooled pasteurized milk with other ingredients (sugar and starter) before processing the yogurt fermentation. The HPLC phenolic profile showed higher phenolic content in OP extract, i.e., gallic acid (1,702.65), chlorogenic acid (1,256.22), naringenin (6,450.57), catechin (1,680.65), and propyl gallate (1,120.37) ppm with massive increases over MP (1.34–37 times) and LP (1.49–5 times). The OP extract successfully scavenged 87% of DPPH with a relative increase of about 16 and 32% over LP and MP, respectively. Additionally, it inhibits 77–90% of microbial growth at 5–8 μg/mL while killing them in the 9–14 μg/mL range. Furthermore, OP extract successfully reduced 77% of human breast carcinoma. Each of pomace powder sample (OP, MP, LP) was added to yogurt at three levels; 1, 3, and 5%, while the physiochemical, sensorial, and microbial changes were monitored during 21 days of cold storage. OP yogurt had the highest pH and lowest acidity, while LP yogurt recorded the reverse. High fat and total soluble solids (TSS) content are observed in OP yogurt because of the high fiber content in OP. The pH values of all yogurt samples decreased, while acidity, fat, and TSS increased at the end of the storage period. The OP yogurts 1 and 3% scored higher in color, flavor, and structure than other samples. By measuring the microbial load of yogurt samples, the OP (1 and 3%) contributes to the growth of probiotics (*Lactobacillus* spp) in yogurt samples and reduces harmful microbes. Using citrus pomace as a source of polyphenols and fiber in functional foods is recommended to enhance their physiochemical and sensory quality.

## Introduction

Even though adults should consume 25–38 g of fiber daily (1), most commonly consumed foods are low in dietary fiber (DF). In this case, processed fruit pomace is usually dumped in a landfill. Adding it to food could have extra health benefits and make the food supply chain more sustainable (2, 3). The food industry annually produces about 4,000 tons of pomace, while raw material mass produces about 25–35% of fruit (4). The majority of pomace is disposed of in landfills, with just a small portion utilized for animal feed and land fertilizer. Because of its high organic matter composition, the disposal of fruit pomace causes environmental pollution (5). Fruit pomace has a high water content, making it an ideal substrate for microbial populations (4).

“Citrus” is a common term for plants of the *Rutaceae* family. It is a popular fruit worldwide, accounting for one-third of the processed yield (6). Byproducts of citrus processing are a rich source of naturally occurring phenolic compounds and fibers. Pectin is abundant in DF. There is an increasing interest in pectin because of its potential to lower blood cholesterol levels and triglycerides; pectin also affects glucose metabolism by lowering the glucose response curve. The major use of pectin is as a food additive because of its specific gelling properties (7). Total dietary fiber content has been determined in different types of citrus fruits. It ranged between 40 and 69 g/100 g DM (8). Most fruit pomace powders have a high SDF content, which causes changes in product attributes such as water solubility and absorption indexes, starch digestibility, texture, expansion, and sensory qualities (9). Dietary fibers may be called prebiotics and are “*a non-digestible food material.”* They influence the host by specifically boosting the development and activity of gut bacteria, thus enhancing host health (10). The extract of citrus fruit pomaces contains many phytochemicals, including phenolic acids and flavonoids, which have antioxidant, antimicrobial, and antitumor activity (6, 11).

Citrus pomaces are rich in bioactive components with good technological and nutritional qualities. Because of their low cost and high value, these wastes can be included as ingredients or additives in food formulations (11, 12). Sugars, flavonoids, carotenoids, folic acid, vitamin C, pectin, and volatile oils are natural compounds found in citrus fruits that are valuable to the food industry and human health. Consequently, citrus fruit pomaces are a great source of polyphenols, which may be isolated and used as natural antioxidants to mitigate oxidation in particular meals and create functional foods (11). Functional foods provide nutrients and bioactive components that can benefit health and reduce the risk of disease in the body (13, 14).

Fermented foods belong to the current category of functional foods. Probiotics have been shown to benefit the immune system and the gut, reduce antibiotic side effects, alleviate symptoms of irritable bowel syndrome, aid in reducing lactose intolerance, and have antimicrobial and anticancer properties (15). Therefore, the consumption of probiotic products treats many health problems such as diarrhea, reduction of lactose intolerance symptoms, reduction of cholesterol in the blood, treatment of irritable and inflammatory bowel diseases, anti-carcinogenic properties, synthesis of vitamins, and enhancing immunity (13). The widespread consumption of yogurt has provided an opportunity to create an extremely valuable functional food item fortified with bioactive components such as vitamins and minerals (16). As a result, daily diets can become more effective in reducing diseases associated with nutritional deficiencies and alleviating the accumulation of bioactive food waste. Yogurt is a precious source of protein and calcium, but it lacks vitamin C and iron, like all dairy products. Yogurt is widely palatable to consumers because of its high nutritional value; therefore, it is a vehicle for different nutrients. Adding prebiotics to yogurt can enhance the probiotics' survival rate and provide additional health benefits to the host (10).

Adding pomace fibers from several sources to the diary increases the product yield, decreases lipid retention, improves textural characteristics and structure, and lowers calorie content because of its water-holding capacity and bulking ability (9). Citrus pomaces can be used as sources of functional compounds and preservatives for newer food products (17). Fiber-rich foods provide significant health benefits (18). Despite the danger of citrus pomace to the environment and health, it has not received the necessary studies. Therefore, in this study, to valorize the citrus pomace from orange, mandarin, and lemon juices, the phenolic profile of these pomaces was detected, and antioxidant, antimicrobial, and anticancer activities were investigated in the pomace extract. Then the changes in yogurt quality after supplementation with citrus pomaces were monitored.

## Materials and methods

### Preparation of orange, mandarin, and lemon pomace aqueous extracts

The orange, mandarin, and lemon pomaces (OP, MP, and LP) were dried in an air oven at 45°C for 3 days, then ground to a fine powder using a Moulinex grinder (France). The extract was prepared as per Saad et al. (19), Ten grams of pomace powder were stirred with 100 mL of distilled water for 3 h at 45°C. Ten grams of pomace powder were stirred with 100 mL of distilled water for 3 h at 45°C. The mixture was filtered, and the soluble fraction was served for physiochemical and antimicrobial analysis. Citrus pomace samples were mixed with methanol/acetone/water (7:7:6, v/v/v) at a ratio of 1:15 g/mL and ultrasonicated at room temperature for 30 min to evaluate the soluble-bound (esterified and glycosylated) phenolics and insoluble-bound phenolics in citrus pomaces. The extracts were centrifuged at 1,200 rpm at 4°C for 10 min. The supernatants were suspended, and the residue was extracted twice more with ethyl acetate under the same conditions (20). Esterified phenolics: The aqueous phases were mixed with 4 M NaOH, 10 mmol/L EDTA and 1% vitamin C at a ratio of 1:2 (v/v) and hydrolyzed at room temperature and 150 rpm for 4 h. The esterified phenolics were obtained as described above in free phenolics. Glycosylated phenolics: The aqueous phases were mixed with 5 mL of 6 mol/L HCl and hydrolyzed at 75°C and 150 rpm for 60 min. The mixtures were extracted with ethyl acetate (1:1, v/v), and glycosylated phenolics were obtained as described above in free phenolics. Insoluble-bound phenolics: The collected residues were mixed with solvent B at a ratio of 1:20 (w/v) and hydrolyzed in a gas bath shaker at room temperature and 150 rpm for 4 h. The insoluble-bound phenolics were obtained as described above in free phenolics.

### Physiochemical properties of citrus pomace

Moisture, protein, fat, and ash contents were estimated as described in AOAC (21). TDF, soluble (SDF), and insoluble (IDF) dietary fibers were determined using McCleary enzymatic and gravimetric methods, McCleary et al. (22). Differential analysis was used to determine the solubility of carbohydrates, and each analysis was conducted in triplicates. The water holding capacity (WHC) was determined as described by Namir et al. (23). In weighted test tubes, 1 g of OP, MP, and CP was homogenized in 10 mL of distilled water, stirred for 30 min, and then centrifuged at 6000xg for 30 min. The supernatant was discarded, and the residues were weighed. WHC was calculated as mL of retained water/g of the sample.

### Phenolic content in orange, mandarin, and lemon pomace aqueous extracts

#### Total phenolic content

The TPC (μg GAE/mL) in citrus pomaces (OP, MP, and LP) extracts were read at 760 nm using a microtiter plate reader (BioTek Elx808, USA) with the modified Folin-Ciocalteu method (24). The absorbance was applied in gallic acid liner equation, y = 0.005x + 0.1455.

#### HPLC free phenolic profile of orange, mandarin, and lemon pomace aqua extracts

The HPLC Shimadzu series (Shimadzu-prominence-20A, Japan) was used to detect the phenolic profile in citrus pomace. The stationary phase was a separation column (Gemini, C18) with a 2 ml/min flow rate (4.6 × 150 mm × 5μm), and the mobile phase was 0.01% acetic acid in water (A) and acetonitrile (B). For the first 5 min, isocratic elution was 95% A/5% B and liner gradient to 50% A/50% B within 5–55 min, then Isocratic elution was 50% A/ 50% B within 55–65 min, and liner gradient to 95% A/5% B at 65–67 min; post-time was 6 min before the next injection. The HPLC pumps, autosampler, column, oven, and diode array system were monitored and controlled. The chromatographic data were processed using Class VP software (Shimadzu 5.0). The phenolic compounds were performed at 280 nm and flavonoids at 370 nm (25, 26).

### Biological activity of orange, mandarin, and lemon pomaces' aqueous extracts

#### Antioxidant

The 2,2-diphenyl-1-picrylhydrazyl (DPPH) radical scavenging assay was used to estimate the antioxidant activity of studied citrus pomace extracts following Saad et al. (19). The percentage of DPPH^·^ scavenging activity was calculated using the following formula:


$$\begin{array}{l} \%\;DPPH\;scavenging\;activity=\frac{Abs\;control-Abs\;sample}{Abs\;control}\times 100 \end{array}$$

The IC50 of aqueous extracts of citrus pomace was estimated as the lowest concentration (μg/ml), which scavenges 50% of DPPH radical.

#### Antimicrobial

Antimicrobial activity of aqueous extracts of OP, MP, and LP was carried out using six pathogenic bacterial strains: *Listeria monocytogenes* (LM), *Bacillus cereus* (BC), *Staphylococcus aureus* (SA), *Yersinia enterocolitica* (YE), *Campylobacter jejuni* (CJ), and *Escherichia coli* (EC) and six fungal strains, *Candida glabrata* (CG), *Candida rugosa* (CR), *Candida stellata* (CS), *Penicillium crustosum* (PC), *Aspergillus niger* (AN), and *Aspergillus flavus* (AF). These strains were selected based on the microbial count of spoiled yogurt samples. It was found during microbial examination with a light microscope and biochemical and morphological definitions that these isolates are the most isolates that cause yogurt spoilage. These isolates were confirmed by identification at the gene level through isolating DNA and using PCR to detect genes. The bacterial isolates were identified based on 16S rRNA and the fungal isolates on 18S rRNA gene sequence analysis. This analysis was carried out at Sigma Scientific Services Co., Giza, Egypt. Sequencing was performed via the automated DNA sequencer (ABI Prism 3130 Genetic Analyzer by Applied Biosystems Hitachi, Japan). Genomic DNA was obtained by the hexadecyltrimethylammonium bromide (CTAB) technique, and the integrity and level of purified DNA were established by agarose gel electrophoresis. The DNA level was customized to 20 ng/μl for PCR amplification. The forward primer used with the isolates is (5 AGA GTT TGA TCC TGG CTC AG 3), and the reverse is (5 GGT TAC CTT GTT ACG ACT T 3). PCR products were isolated by electrophoresis on 1.5% agarose gels stained with ethidium bromide and documented in the Alphaimager TM1200 documentation and analysis system. The obtained polymorphic differences were analyzed via the program NTSYS-PC2 by assessing the distance of isolates by Jaccard's Similarity Coefficient. The antimicrobial activity was carried out using the disc diffusion method (27); the plates were incubated at 37°C overnight (in the case of bacteria) and 28°C for 5 days in the case of fungi. The inhibition zones were recorded in mm for replicates prepared for each treatment. The values of minimum inhibitory concentration (MIC), minimum bactericidal concentration (MBC), and minimum fungicidal concentration (MFC) were evaluated as per El-Saadony et al. (16).

#### Anticancer

The sulphorhodamine B (SRB) test was used to determine MCF-7 cell line viability. In 96-well plates, 100 μl of cell suspension (5 × 10^3^) was placed in control media and incubated for 24 hours. Another 100 mL was placed in the medium supplemented with different concentrations of citrus pomace (10, 20, 30, 40, and 50 μg/mL) under the same conditions. The cells were fixed for 72 h by adding 150 μL of 10% trichloroacetic acid (TCA) and incubated at 4°C for 1 h. The cells were washed several times with distilled water. Afterward, 70 μL of SRB solution (0.4% w/v) was added and incubated in the dark for 10 min. Plates were washed three times with 1% acetic acid and air-dried overnight. 150 μL of TRIS-HCl (10 mM) was added to dissolve the protein-bound SRB stain; the absorbance was read at 540 nm using a microtiter plate reader (BioTek Elx808, USA).

### Processing of yogurt supplemented with orange, mandarin, and lemon pomace powder

The starter culture (Christian Hansen laboratories) was prepared from 1 g of each lyophilized probiotic (1:1:1) “*Lactobacillus acidophilus* LA-5 (containing 1.5 × 10^8^ CFU/g), *Bifidobacterium Bb12* (containing 1.0 × 10^7^ CFU/g), and *Streptococcus thermophilus”* (containing 1.5 × 10^7^ CFU/g). The final concentration of strater culture was 1%.

The yogurt preparation was done as the milk base was heated at 85°C for 30 min, and then cooled to 42.5°C. Yogurt culture was added at 1%, then the powder of LP, MP, and OP at three levels (1, 3, and 5%) were also added to the milk and 10% of sugar, and the fermentation was proceeded at 42.5°C for 5h until a firm curd was formed. Then the yogurt was cooled down to 4°C and stored for 12 h. The samples were mixed for 50 s with an electric stirrer before being packed in sterilized cups and stored at 4°C in the refrigerator.

### Fluctuation during yogurt preservation period

#### Physiochemical properties

The pH values of treated and untreated yogurt samples were estimated by pH meter (pH 211 HANNA, Romania). The titratable acidity was estimated by the standard method 942.15 and expressed as lactic acid (%) at the cold preservation period (0–21 days). The TSS were estimated by a refractometer. Also, vitamin C content was determined as AOAC (21). Fat content was measured by the Gerber method;increasing fat content within the range 0.37 to 4%, while maintaining the protein constant, resulted in enhancing texture, stability and apparent viscosity.

#### Color analysis and sensory properties

Hunter Lab spectrophotometer (Color Flex EZ's, USA) was used to analyze the color parameters of yogurt samples, L^*^, a^*^, b^*^, to calculate the color change ΔE following Namir et al. (18).

Ten trained professional panelists (6 males and 4 females, aged 40–55 years) evaluated the sensorial properties of yogurt samples. The following attributes were evaluated: flavor, color, texture, shape, and over-acceptability). Each member was given water to wash away the effects of each sample, which was accordingly judged (16).

#### Microbial changes during yogurt preservation

10 g of treated and untreated yogurt samples were mixed in 90 mL of peptone buffer water to prepare a suspension. Serial decimal dilutions of 10^−1^–10^−8^ were prepared. The dilutions The dilutions placed in one-use Petri-dishes supplemented with certain media (28). Plate count agar was used to enumerate the total bacterial count (TBC) after incubation at 30°C for 24 h. Lactic acid bacteria (LAB) were enumerated at MRS medium after incubation at 37°C for three days (16). The proteolytic bacteria count was enumerated following Celik et al., (29). Additionally, lipolytic bacteria were counted following Gu, Xing Li (30). Microbial results were altered to logarithms (CFU/g).

### Statistical analysis

The data were analyzed using Microsoft Office Excel (V.2019) through one-way ANOVA variance. The least significant difference (LSD) was used as a *post-hoc* test to elucidate the differences between means.

## Results and discussion

### Physiochemical composition of orange, mandarin, and lemon pomaces

The chemical composition, functional, and color properties of citrus pomaces, OP, MP, and LP, are listed in Table 1. The TDF was the most abundant component of citrus pomaces, ranging from 71–77 g/100 g depending on the citrus kind. The OP has higher TDF content than MP and LP. The TDF content of citrus pomace was significantly higher than that of citrus peels (57 g/100 g) (31) but was comparable to that of passion fruit byproduct and apple pomace (81.50 and 82.00 g/100 g, respectively) (32). Insoluble dietary fibers (IDF) comprise 51.5258.11% of TDF in citrus pomace; soluble dietary fibers (SDF) comprise 18.9120.55% of TDF in AIR-PPB. In general, fruit and vegetable byproducts are high in dietary fiber and soluble sugars, but low in fat and protein (33), and the dietary fibers were combined with low protein, low fat, and soluble carbohydrates in this extraction method.

**Table 1:** Physiochemical composition, water holding capacity (WHC), and color attributes of citrus pomace powder.

**Composition (g/100 g)**	**Citrus pomace**		
	**OP**	**MP**	**LP**
**Chemical**	
Moisture (w.b)	5.32 ± 0.04a	5.67 ± 0.06b	5.61 ± 0.00bc
Protein	5.67 ± 0.11a	5.40 ± 0.18a	5.38 ± 0.02ab
Fat	0.61 ± 0.01a	0.77 ± 0.00ab	0.89 ± 0.01ab
Ash	4.11 ± 0.24a	5.33 ± 0.02ab	7.22 ± 0.11bc
Soluble carbohydrates	6.09 ± 0.29d	6.50 ± 0.27d	6.63 ± 0.35c
Dietary Fibers			
TDF	77.02 ± 0.40a	73.91 ± 0.35b	71.87 ± 0.11c
IDF	58.11 ± 0.53a	53.80 ± 0.35b	51.52 ± 0.44c
SDF	18.91 ± 0.11e	20.11 ± 0.10d	20.55 ± 0.09c
**Physical**	
WHC (ml/g)	7.77 ± 0.20a	7.65 ± 0.81b	7.19 ± 0.34c
L*	63.22 ± 0.71e	65.14 ± 0.00d	68.79 ± 0.63c
a*	14.52 ± 0.68a	12.65 ± 0.53b	10.45 ± 0.00c
b*	46.28 ± 0.23e	50.75 ± 0.00d	53.36 ± 0.21c

w.b, wet basis; TDF, total dietary fibers; IDF, insoluble dietary fibers; SDF, soluble dietary fibers. The triplicate data are expressed as mean ± SD. Means within a row with different lowercase letters are significantly different at the level of p < 0.05. L^*^, whiteness; a^*^, redness, b^*^, yellowness'.

The WHC of citrus pomace ranged 7.19–7.77 mL/g, compared to 5.70, 4.90, and 4.12 mL/g for the date, pear, and tomato pomaces, respectively. Additionally, the WHC of the cantaloupe byproduct was 6.17 mL/g (34). The hydroxyl groups in polysaccharide chains may form hydrogen bonds with water, thereby increasing the water-holding capacity of fiber-rich materials (35). The lower WHC values in citrus pomace can be attributed to the degradation of polysaccharide chains, increasing the soluble fibers fraction.

Table 1 demonstrates a significant increase in the values of lightness and yellowness in citrus pomace but a significant decrease in redness values. Industrial byproducts can differ considerably in their chemical composition. Additionally, it is important to consider that certain byproducts require special treatments and processing prior to further utilization due to their chemical composition. These byproducts' chemical composition and structure significantly impact their functional properties and applications. Additionally, byproducts are naturally colored differently. Therefore, high levels of these byproducts may cause color changes in food formulations, which may or may not be desired by consumers (35).

### Antioxidant content of aquas extract of orange, mandarin, lemon pomaces

#### Antioxidant activity

The phenolic content indicates the antioxidant capacity and is a preliminary screen for any product supposed to be employed as a natural source of antioxidants in functional foodstuffs (36). Several studies investigated the relationship between phenolic content and antioxidant activity in various plants and fruits. The antioxidant mechanism of phenolic compounds may be related to their high redox potentials, which permit them to act as reducing agents, hydrogen donors, and single oxygen scavengers (37).

**Figure 1:**
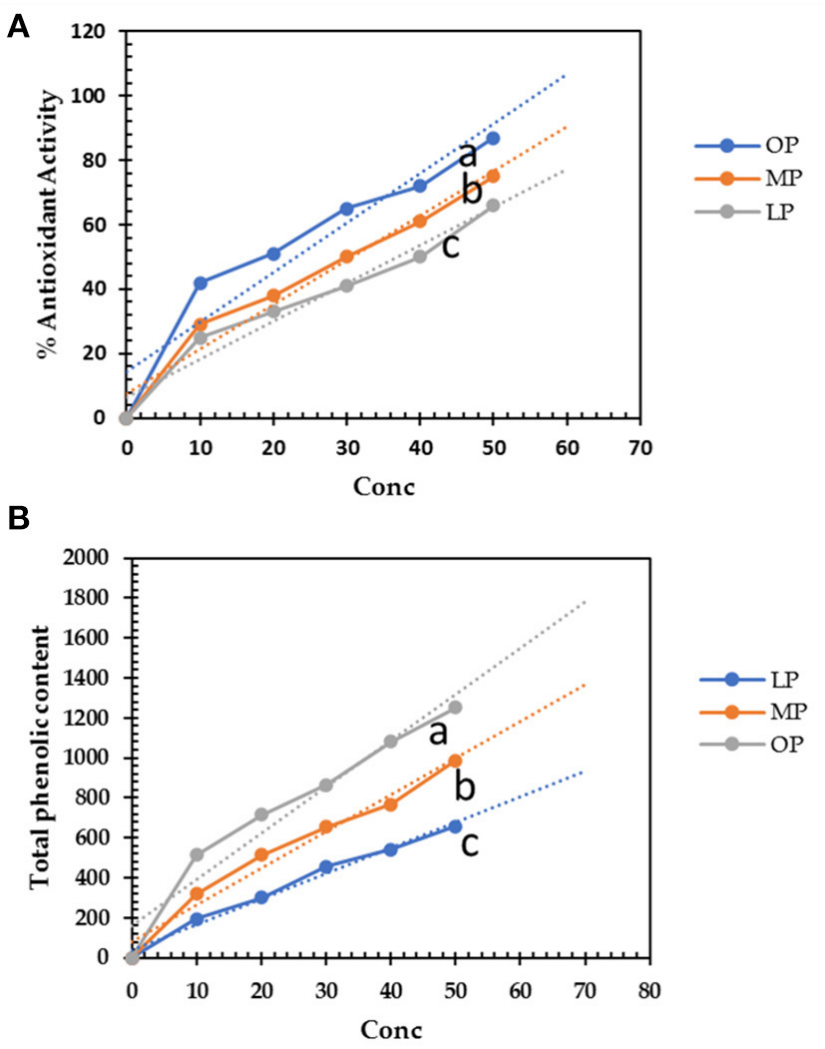
**(A)** Total phenolic content (mg gallic acid equivalent/g) of lemon, mandarin, orange pomaces powder aquas extracts (10-50 μg/mL). **(B)** DPPH^·^ scavenging activity of lemon, mandarin, orange pomaces powder aquas extracts. Lowercase letters besides curves indicate signficant differences.

The aqueous extracts of LP, MP, and OP exhibit considerable antioxidant activity. OP extract (50 μg/mL) successfully scavenged 87% of DPPH radical and was expected to increase the scavenging activity in concentration dependence (Figure 1A). The antioxidant activity of OP (50 μg/mL) increases by 16 and 32% compared to LP and MP, respectively. The SC50 of OP, MP, and LP was 20, 30, and 40 μg/mL, respectively. The antioxidants are attributed to the phenolic content of these extracts. OP extract scored the highest phenolic content with a relative increase of about 28 and 90 % over LP and MP extracts, respectively (Figure 1B). The use of water for extraction was safe without any residues of chemical solvents. Second, it was proved that aqueous extract of pomaces generally was more efficient than ethanolic one; in Wang et al., (38) study, it was found that citrus pomace water extract (CPW) contained 3.35 ± 0.00 g/100 g GAE phenolic content and 4.07 ± 0.73 g/100 g RE flavonoid content, and scavenged DPPH, alkyl, and hydroxyl radicals at the IC50 of 0.16 ± 0.00, 0.31 ± 0.01, and 0.86 ± 0.02 mg/mL, respectively and that more efficient than citrus pomace ethanolic extract (CPE) which reported that 70% ethanol extract of CP (CPE) contained 2.69 ± 0.10 g/100 g GAE phenolic content and 1.27 ± 0.46 g/100 g RE flavonoid content, respectively. In addition, CPW scavenged DPPH, alkyl, and hydroxyl radicals at the IC50 of 0.65 ± 0.02, 0.33 ± 0.02, and 0.67 ± 0.01 mg/mL, respectively (39). These results show that CPW contains higher phenolic and flavonoid contents and possesses stronger DPPH and alkyl scavenging activities than CPE.

The free phenolic compounds were 1,108, 930, and 581 mg/g for OP, MP, and LP, respectively (Figure 1B), but the soluble-bounded phenolic contents were 250, 190, and 99 mg/g for OP, MP, and LP, respectively and insoluble-bounded estimated by 123, 85, and 56 mg/g for OP, MP, and LP. Yao et al. (20) found that the total phenolic contents of different phenolic fractions from raspberry pomace by the Folin method followed the order: free > esterified ≈ insoluble-bound > glycosylated. Thus, depends on extraction methods, solvents, and the bounded compounds, mainly dietary fibers.

According to Fernández et al. (40), the phenolic content of mandarin pomace ranged from 6.6 to 11.0 mg GAE/g, whereas grapefruit, orange, and lemon peels had high phenolic content, i.e., 77.3, 49.8, and 35.6 mg of GAE/g, respectively, with a moderate antioxidant activity ranging from 73 to 75% (41).

The capacity of phenolic compounds to scavenge free radicals, eliminate radical chain reactions, and chelate metals is associated with their antioxidant activity (42). While the antioxidant activity of plant extracts depends on their chemical structure and antioxidant content, Antioxidants are extensively used as food additives to help foods degrade and enhance shelf life by reducing lipid peroxidation and preventing oxidative damage (43).

#### HPLC soluble phenolic profile of aquas extracts of fresh orange, mandarin, lemon pomaces

Table 2 shows the free phenolic profile of aqueous extracts of OP, MP, and LP. The HPLC profile shows phenolic acids, polyphenols, and flavonoids. Higher phenolic content was recorded in OP extract, i.e., gallic acid (1,702.65), chlorogenic acid (1,256.22), naringenin (6,450.57), catechin (1,680.65), and propyl gallate (1,120.27) ppm with massive increases over MP (1.34–37 fold) and LP (1.49–5 fold). Additionally, medium phenolic content such as ferulic acid, rutin, quercetin, and catechol with 630.24, 1,090.29, 460.57, and 565.33 ppm, respectively, other polyphenols are of low content; however, vanillin was in traces. Naringenin and catechin were high in MP. However, ellagic acid and vanillin were low. On the other hand, LP extract lacks chlorogenic acid, syringic acid, caffeic acid, and caffeine. Citrus peels have high phenolic and flavonoid content, which exhibit considerable antioxidant capacity. Flavonoids and flavanols have various biological activities, including radical scavenging (17, 44). Montenegro-Landvar et al. (45) that aqueous extract of orange waste might be a substantial source of 4-hydroxybenzoic acid and hesperidin. The flavonoid contents in the citrus pomace's methanolic extract showed nobiletin, hesperidin/neo-hesperidin, tangerine, heptamethoxyflavone, tetramethyl scutellarin, and naringin/narirutin were detected by UHPLC-MS/MS (40).

**Table 2:** HPLC determined free phenolic compounds (ppm) of lemon, mandarin, orange pomaces powder aquas extracts expressed as (mean ±SD).

**Phenolic compounds**	**Conc (ppm)**		
	**LP**	**MP**	**OP**
Gallic acid	855.9 ± 0.01^aC^	988.5 ± 0.5^cB^	1,702.65 ± 0.6^bA^
Chlorogenic acid	5.33 ± 0.00^jC^	530.44 ± 0.3^dB^	1,256.22 ± 0.1^bcA^
Cinnamic acid	21.55 ± 0.12^iB^	25.33 ± 0.01^iA^	22.68 ± 0.8^hB^
Syringic acid	4.55 ± 0.32^jC^	100.36 ± 0.0^hB^	210.45 ± 0.09^eA^
Caffeic acid	4.33 ± 0.65^jC^	105.33 ± 0.6^hA^	55.22 ± 0.1g^hB^
Ellagic acid	106.33 ± 0.1^fB^	5.65 ± 0.3^jC^	250.33 ± 0.2^eA^
Coumaric acid	60.33 ± 0.09^gB^	65.45 ± 0.45^hiB^	81.66 ± 0.3^gA^
Ferulic acid	75.67 ± 0.08^gC^	270.55 ± 0.1^gB^	630.24 ± 0.0^cA^
Rutin	320.58 ± 0.2^cC^	701.54 ± 0.2^dB^	1,090.29 ± 0.0^bcA^
Vanillin	35.67 ± 0.3^hA^	7.24 ± 0.1^jC^	15.33 ± 0.3^hB^
Naringenin	170.25 ± 0.01^eC^	3,945.3 ± 0.02^aB^	6,450.57 ± 0.2^aA^
Catechin	501.39 ± 0.00^bC^	1,250.12 ± 0.06^bB^	1,680.65 ± 0.11^bA^
Querectin	49.21 ± 0.9^hC^	340.22 ± 0.5^fB^	460.57 ± 0.2^dA^
Caffeine	6.25 ± 0.8^jC^	35.84 ± 0.7^iB^	125.78 ± 0.34^fA^
4‘.7-DihydroxyisoFlavone	110.65 ± 0.2^fB^	112.3 ± 0.3^hB^	205.64 ± 0.5^eA^
Propyl Gallate	230.47 ± 0.1^dC^	455.87 ± 0.2^eB^	1,120.27 ± 0.6^bcA^
Catechol	120.85 ± 0.4^fC^	302.44 ± 0.4^fB^	565.33 ± 0.7^cA^
P-OH-benzoic	211.54 ± 0.5^dB^	250.65 ± 0.3^gB^	325.79 ± 0.01^dA^

Means with different lowercase letters in the same column indicate the differences between phenolic compounds in one pomace extract. Different uppercase letters in the same row indicate significant differences between citrus pomace p < 0.05.

### Antimicrobial activity

Table 3 shows the results of the inhibition zones of citrus extracts against microbial strains. The highest antibacterial activity was obtained with OP against *B. cereus* (BC) and *S. aureus* (SA), with inhibition zone diameters of 27 and 25 mm, respectively. The lowest antibacterial activity was against *Y. enterocolitica* (YE) and *C. jejuni* (CJ) with inhibition of 22- and 21-mm. OP also exhibited considerable antifungal activity with large inhibition zones of 28 and 29 mm against *P. crustosum (PC)* and *A. flavus* (AF) and the lowest inhibition zones of 21 and 25 mm against *C. glabrata* (CG) and *A. niger* (AN). The effectiveness of the extracts can be summarized by an aqueous extract of OP > MP > LP.

**Table 3:** Inhibition zones diameters (mm) of lemon, mandarin, orange pomaces powder aquas extracts against milk pathogenic microorganisms (mean ± SD).

**Pathogenic bacteria**	**LP (**μ**g/mL)**				**MP(**μ**g/mL)**				**OP(**μ**g/mL)**			
	**5**	**10**	**30**	**50**	**5**	**10**	**30**	**50**	**5**	**10**	**30**	**50**
*L. monocytogenes (LM)*	ND	11 ± 0.2b	13 ± 0.1c	19 ± 0.7b	ND	12 ± 0.3c	15 ± 0.1cd	20 ± 0.3c	9 ± 0.2b	14 ± 0.1cd	19 ± 0.1c	22 ± 0.6c
*B. cereus (BC)*	ND	12 ± 0.4ab	15 ± 0.4b	20 ± 0.9b	8 ± 0.01a	14 ± 0.5b	17 ± 0.2b	21 ± 0.3b	10 ± 0.1ab	16 ± 0.9b	20 ± 0.3b	25 ± 0.7b
*S. aureus (SA)*	8 ± 0.0a	14 ± 0.1a	18 ± 0.3a	23 ± 0.3a	9 ± 0.03a	16 ± 0.2a	21 ± 0.4a	25 ± 0.2a	11 ± 0.4a	18 ± 0.8a	23 ± 0.2a	27 ± 0.5a
*Y. enterocolitica (YE)*	ND	9 ± 0.3d	13 ± 0.4c	17 ± 0.4cd	ND	12 ± 0.5cd	16 ± 0.6c	20 ± 0.1c	ND	14 ± 0.3cd	17 ± 0.6d	22 ± 0.5c
*C. jejuni (CJ)*	ND	8 ± 0.5d	12 ± 0.6d	15 ± 0.5d	ND	10 ± 0.5d	15 ± 0.7cd	19 ± 0.2d	ND	12 ± 0.1d	16 ± 0.9d	21 ± 0.3cd
*E. coli (EC)*	ND	10 ± 0.3c	15 ± 0.3b	18 ± 0.6c	ND	13 ± 0.3c	170.3 ± b	21 ± 0.4b	8 ± 0.0c	15 ± 0.3c	18 ± 0.3cd	24 ± 0.1bc
**Pathogenic fungi**	**LP(**μ**g/mL)**				**MP(**μ**g/mL)**				**OP(**μ**g/mL)**			
*C. glabrata (CG)*	ND	12 ± 0.1d	13 ± 0.0e	16 ± 0.3d	ND	14 ± 0.2c	17 ± 0.6c	21 ± 0.3c	ND	17 ± 0.3c	19 ± 0.1c	21 ± 0.2cd
*C. rugosa (CR)*	ND	16 ± 0.5b	18 ± 0.1b	21 ± 0.6b	ND	17 ± 0.3b	20 ± 0.9b	23 ± 0.5b	12 ± 0.2c	20 ± 0.1b	22 ± 0.2b	25 ± 0.2bc
*C. stellata (CS)*	ND	16 ± 0.3b	19 ± 0.5a	23 ± 0.4a	11 ± 0.2b	18 ± 0.6ab	22 ± 0.4a	25 ± 0.7ab	13 ± 0.1b	21 ± 0.6ab	24 ± 0.5ab	27 ± 0.4b
*P. crustosum (PC)*	11 ± 0.2a	17 ± 0.2a	18 ± 0.3b	20 ± 0.4b	13 ± 0.3a	19 ± 0.7a	22 ± 0.3a	26 ± 0.9a	15 ± 0.3a	22 ± 0.3a	25 ± 0.3a	29 ± 0.3a
*A. niger (AN)*	ND	14 ± 0.9c	16 ± 0.2d	19 ± 0.7c	ND	16 ± 0.3bc	18 ± 0.1c	20 ± 0.6c	ND	18 ± 0.2c	21 ± 0.8bc	25 ± 0.1bc
*A. flavus (AF)*	ND	15 ± 0.2bc	17 ± 0.1c	20 ± 0.3b	ND	18 ± 0.1ab	20 ± 0.4b	22 ± 0.1b	14 ± 0.6ab	21 ± 0.6ab	25 ± 0.3a	28 ± 0.3ab

ND, not detected. Means with different lowercase letters in the same column indicate significant differences p < 0.05.

Sharma et al., (46) observed that Citrus wastes have antibacterial activity. The mandarin waste had higher antimicrobial activity than lemon waste, as demonstrated by Espina et al. (47). The antimicrobial activities of citrus are related to flavonoids and phenols (48). The aqueous extract of the lemon fruit contained phytochemicals such as alkaloids, flavonoids, phenols, quinines, terpenoids, and carbohydrates, which possess a considerable antimicrobial activity against several bacterial species (17). Different studies reported that plant extracts were more effective against gram-positive bacteria than gram-negative bacteria due to an additional lipopolysaccharide coat (49). Our results are in agreement with these studies. Likewise, Min et al. (50) found that unshiu peel extract exhibited antibacterial activity against bacterial strains, which inhibited the growth of *Bacillus cereus, Listeria monocytogenes*, and *Staphylococcus aureus*. Caputo et al. (51) found that hot water extracts of three citrus peels (orange, lemon, and citron) effectively against ten different sanitary-relevant bacteria. Medium antimicrobial activities were detected in citrus waste ethanolic extracts with pressure (52). Oikeh et al. (53) reported that the ethanolic citrus peel extract contains more phenolic and significant antimicrobial activities against the microbial strains, comparing the dry peel extract.

**Figure 2:**
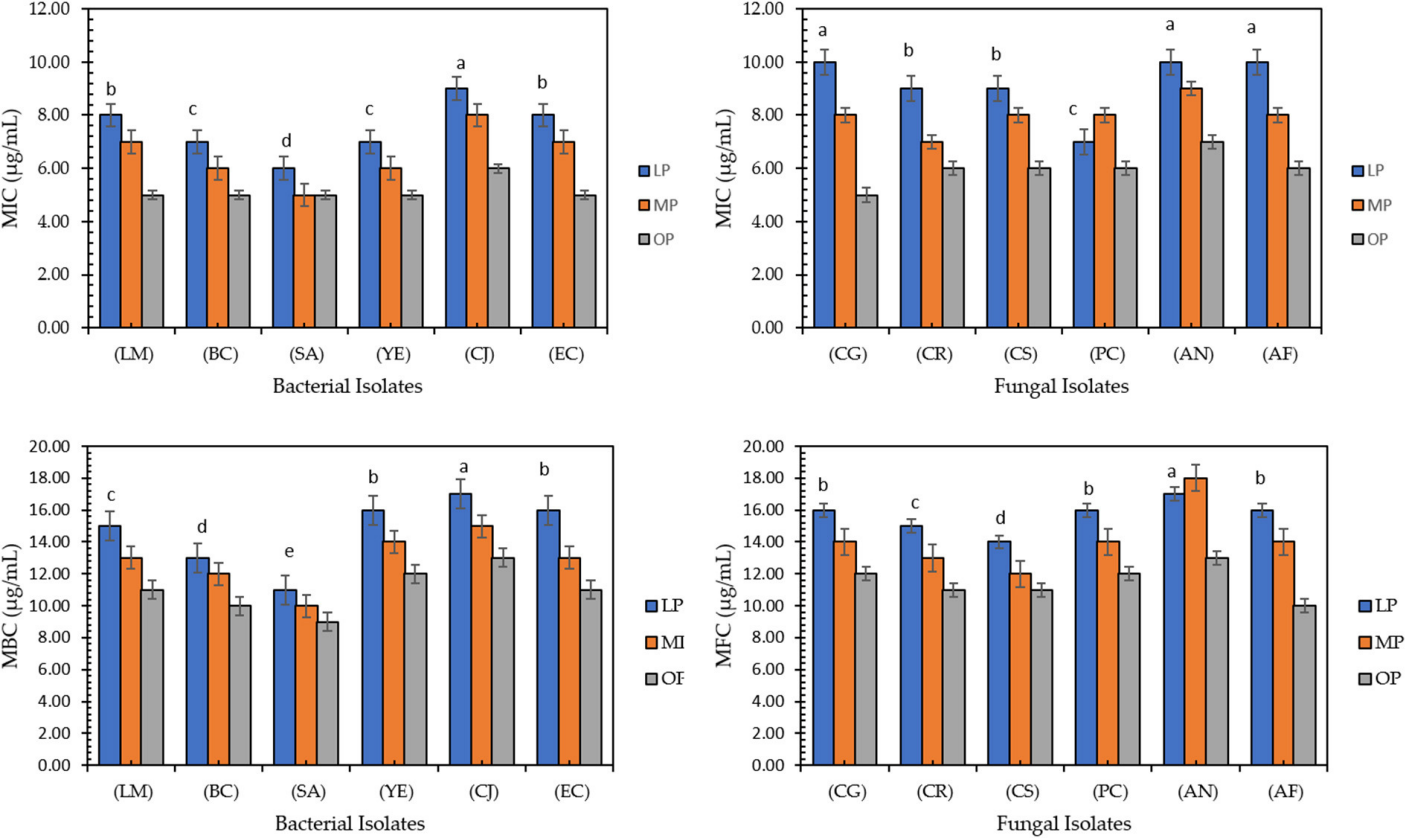
The lowest concentration (μg/mL) of lemon, mandarin, orange pomaces powder aquas extracts inhibiting microbial strains, (MIC), the lowest concentration (μg/mL) killing bacterial strains, (MBC), and fungal strains (MFC). *Listeria monocytogenes* (LM), *Bacillus cereus* (BC), *Staphylococus aureus* (SA), *Yersinia enterocolitica* (YE), *Campylobacter jejuni* (CJ), and *Escherichia coli* (EC) and six fungal strains, *Candida glabrata* (CG), *Candida rugosa* (CR), *Candida stellata* (CS), *Penicillium crustosum* (PC), *Aspergillus niger* (AN), and *Aspergillus flavus* (AF). Data presented mean ±SD, Lowercase letters above column indicate signficant differences.

Figure 2 shows minimum inhibitory concentration, minimum bactericidal concentration, and minimum fungicidal concentration values of citrus pomace extracts against bacterial and fungal strains. The OP extract records the lowest MIC against all bacterial and fungal strains compared to LP and MP; it inhibits the microbial strains in the range of 5–8 μg/mL while killing the microbial strains in the range of 9–14 μg/mL. The highest MIC, MBC, and MFC values were recorded in LP. Fiorentini et al. (54) found that food-grade commercial citrus peel extract (5–0.625 mg/mL) inhibits the growth of *Staphylococcus aureus*, which was maintained after the encapsulation process (MIC 5–1.25 mg/mL).

### Cytotoxicity activity of citrus pomace against MCF-7 cells

**Figure 3:**
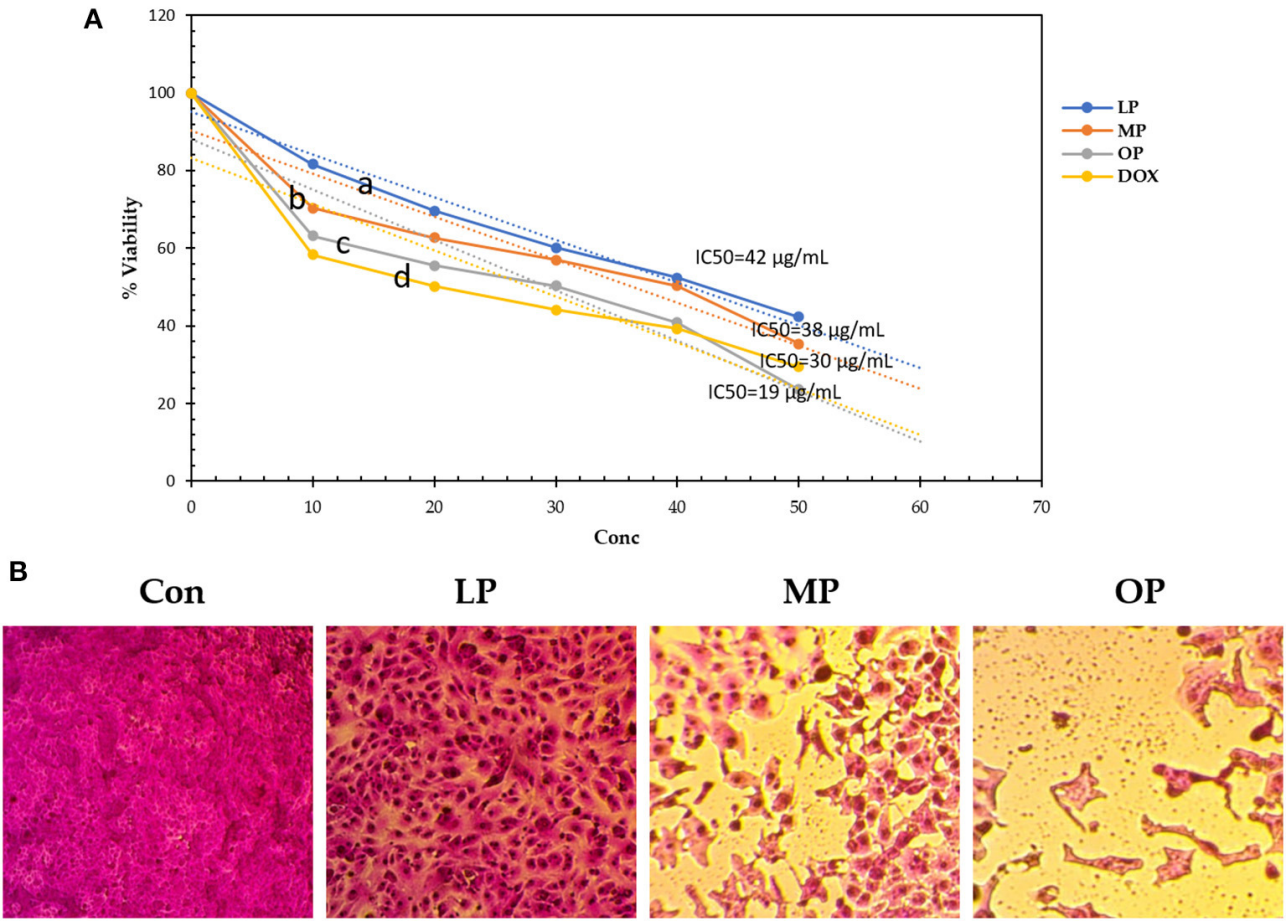
**(A)** The viability of MCF-7 (%) affected by lemon, mandarin, orange pomaces powder aquas extracts **(B)** CON, MCF-7 cell without any addition; LP, MCF-7 cell affected by LP; MP, MCF-7 cell affected by MP; OP, MCF-7 cell affected by OP compared to Doxorubicin. Lowercase letters besides curves indicate signficant differences.

Table 4 and Figure 3 show the IC50 of OP, MP, and LP against breast carcinoma (MCF-7) compared to Doxorubicin. The results revealed that OP, MP, and LP exhibited efficient cytotoxicity against human tumor cell lines representing human breast carcinoma relative to the positive Doxorubicin. OP has the lowest IC50 of 19 μg/mL, compared to MP, LP, and Doxorubicin with 38, 42, and 30 μg/mL, respectively. The OP (50 μg/mL) successfully reduced 77% of human breast carcinoma (live cell of MCF-7 was 23%), while MP, LP, and Doxorubicin reduced 58–71% of MFC-7 cells. The highest dead cell percentage recorded by OP, then MP, then LP may be related to the presence of phenolic compounds such as syringic acid and naringin (Table 1).

**Table 4:** The viability of MCF-7 cells (%) affected by lemon, mandarin, orange pomaces powder aquas extracts (mean ± SD).

**Conc. (μg/mL)**	**%MCF-7 viability**			
	**LP**	**MP**	**OP**	**DOX**
0	100 ± 0.0a	100 ± 0.0a	100 ± 0.0a	100 ± 0.0a
10	81.65 ± 0.2bA	70.34 ± 0.6bB	63.23 ± 0.9bBC	58.35 ± 0.1bC
20	69.56 ± 0.4cA	62.65 ± 0.9cA	55.52 ± 0.3cB	50.23 ± 0.3bcB
30	60.12 ± 0.5cA	56.98 ± 0.2cdB	50.36 ± 0.4cB	44.14 ± 0.6cC
40	52.36 ± 0.9cdA	50.28 ± 0.8cdAB	40.89 ± 0.6dB	39.25 ± 0.8dB
50	42.25 ± 0.3dA	35.33 ± 0.6dB	23.65 ± 0.9eC	29.47 ± 0.3eC

Means with different lowercase letters in the same column indicate the differences between effect of one pomace extract on cancer cell viability. Different uppercase letters in the same row indicate significant differences between citrus pomace p < 0.05.

Apoptosis may be a specialized process of cell death that is a part of the normal development of organs and tissue maintenance but can also occur as a response to varied environmental stimuli, indicating toxicity. Since apoptosis can play a critical role in cancer development, toxins' ability to induce apoptosis appears to be linked to their toxicological effects (55). In this respect, Tajaldini et al. (56) studied the anticancer effects of orange peel aqueous extract against esophageal cancer stem cells. They discovered that combining Doxorubicin and orange peel extract can reduce tumor size and body weight in treated nude mice compared to Doxorubicin alone, showing less systemic toxicity and decreased oxidative stress. Additionally, Wang et al. (38) found a medium reduction of Vero cells by citrus pomace aqueous extract.

### The experiment of yogurt supplemented with lemon, mandarin, and orange pomaces powder

#### Physiochemical parameters

Table 5 presents the possible fluctuations in physiochemical attributes of yogurt supplemented with OP, MP, and LP powders (1, 3, and 5%) during cold storage for 3 weeks. The TSS significantly increased during cold storage in treated yogurt (*p* ≤ 0.05) compared to control, ranging from 12.3 to 16.8. The increment is because yogurt whey evaporated during cold storage (57) or due to the breakdown of lactose into simple sugar (58). The total sugars (soluble and insoluble) were decreased due to the breakdown of total sugar by the fermentative effect of acid-producing bacteria, increasing TSS (26).

**Table 5:** Physicochemical properties of yogurt supplemented with lemon, mandarin, orange pomaces powder (1, 3, and 5%) during cold preservation (mean ± SD).

**Pomace powder**	**Yogurt samples**	**Storage (days)**	**pH**	**Acidity (mg/10mL)**	**Fat (%)**	**TSS (%)**
	Control	0	4.36 ± 0.1aD	91.30 ± 0.2bB	1.30 ± 0.2cD	12.30 ± 0.6bD
		10	4.30 ± 0.1bD	91.98 ± 0.1bB	1.50 ± 0.3bD	13.10 ± 0.6abD
		21	4.28 ± 0.2bD	93.29 ± 0.0aB	1.80 ± 0.6aD	13.90 ± 0.5aD
	Y1%	0	4.39 ± 0.2bC	91.39 ± 0.0dA	1.70 ± 0.5cdC	12.90 ± 0.9dC
LP		10	4.31 ± 0.cC	92.62 ± 0.2cA	1.95 ± 0.3cC	13.60 ± 0.1cC
		21	4.29 ± 0.2bcC	93.76 ± 0.3bA	2.10 ± 0.6abC	14.20 ± 0.2bC
	Y3%	0	4.43 ± 0.1abC	91.58 ± 0.4dA	1.87 ± 0.3cC	13.12 ± 0.1cdC
		10	4.38 ± 0.3bC	93.72 ± 0.2bA	2.00 ± 0.4bC	14.70 ± 0.8bC
		21	4.31 ± 0.6cC	95.05 ± 0.4aA	2.30 ± 0.9aC	15.33 ± 0.6abC
	Y5%	0	4.50 ± 0.7aC	91.92 ± 0.3dA	1.00 ± 0.1eC	13.45 ± 0.5cC
		10	4.45 ± 0.6abC	94.05 ± 0.5abA	1.30 ± 0.2dC	15.20 ± 0.1abC
		21	4.41 ± 0.4abC	95.98 ± 0.6aA	1.40 ± 0.0dC	15.80 ± 0.2aC
	Y1%	0	4.49 ± 0.3bB	88.35 ± 0.9cC	1.90 ± 0.2cdB	13.85 ± 0.3eB
MP		10	4.41 ± 0.5bcB	89.58 ± 0.5cC	2.15 ± 0.0bB	14.55 ± 0.2dB
		21	4.39 ± 0.6cB	90.72 ± 0.1bcC	2.30 ± 0.3bB	15.15 ± 0.0cB
	Y3%	0	4.53 ± 0.4abB	88.54 ± 0.3cC	2.07 ± 0.1cB	14.07 ± 0.0dB
		10	4.48 ± 0.5bB	90.68 ± 0.0bcC	2.20 ± 0.2bB	15.65 ± 0.1cB
		21	4.41 ± 0.9cB	92.01 ± 0.1aC	2.50 ± 0.4aB	16.28 ± 0.3bB
	Y5%	0	4.60 ± 0.0aB	88.88 ± 0.09cC	1.20 ± 0.01eB	14.40 ± 0.4dB
		10	4.55 ± 0.4abB	91.01 ± 0.2bC	1.50 ± 0.05dB	16.15 ± 0.2bB
		21	4.51 ± 0.3abB	92.94 ± 0.1aC	1.60 ± 0.09dB	16.75 ± 0.3aB
	Y1%	0	4.68 ± 0.1bcA	85.32 ± 0.8dD	2.06 ± 0.1dA	14.10 ± 0.4dA
OP		10	4.60 ± 0.7cA	86.55 ± 0.9cD	2.31 ± 0.3cA	14.80 ± 0.3cA
		21	4.58 ± 0.3cA	87.69 ± 0.4bD	2.46 ± 0.9bA	15.40 ± 0.5bA
	Y3%	0	4.72 ± 0.4abA	85.51 ± 0.1dD	2.23 ± 0.5cdA	14.32 ± 0.6cA
		10	4.67 ± 0.3bcA	87.65 ± 0.3bD	2.36 ± 0.6cA	15.90 ± 0.3bA
		21	4.60 ± 0.6cA	88.98a ± 0.6bD	2.66 ± 0.1aA	16.53 ± 0.7abA
	Y5%	0	4.79 ± 0.3aA	85.85 ± 0.6dD	1.36 ± 0.0fA	14.65 ± 0.3cA
		10	4.74 ± 0.9abA	87.98 ± 0.8bD	1.66 ± 0.1efA	16.40 ± 0.8abA
		21	4.70 ± 0.1bA	89.91 ± 0.2aD	1.76 ± 0.3eA	16.88 ± 0.6aA

Means with different lowercase in the same column indicate the difference between physiochemical parameters between different concentration of one yogurt and uppercase letters in the same column indicate significant differences between all yogurt samples p < 0.05.

Because of moisture changes due to citrus pomace concentrations (Table 4), a significant increase in fat content was observed between control and treated yogurt during cold preservation for 3 weeks (*p* < 0.05) (57).

The pH values slightly increased in OP yogurt, while LP yogurt had the lowest pH compared to other samples. LP yogurt had higher acidity than other samples, with 95.98 mg of lactic acid per 10 g. The pH of yogurt samples ranged from 4.3 to 4.7, and acidity as lactic acid was averaged from 85 to 95.98 mg. The pH values (*p* ≤ 0.05) decreased during the cold preservation duration in all yogurt samples. The gradient concentration of citrus pomace did not affect the incubation time required for the yogurt mixes to reach pH 4.3, and the starter culture populated and produced lactic acid that decreased pH and increased acidity. These findings agree with those of Arioui et al. (59) and Salehi (60) (Table 4). Many studies have found that the rheological qualities of yogurt vary depending on the type and source of fiber (33). Fibers' abilities to increase water holding capacity, stabilize high-fat yogurt, enhance viscosity characteristics, and gel-forming ability enable the production of fiber-enriched yogurt with improved texture and reduce syneresis (61).

#### Sensory properties and color changes

Sensory evaluation of yogurt supplemented with different concentrations of OP, LP, and MP powder during cold storage periods at (4°C) after 21 days is shown in Table 6. The results confirmed that the additional levels of 1 and 3% of OP, LP, and MP powder to fermented milk possessed the best flavor, with no significant difference in between, but significantly differed in comparison with control and 5% of OP, LP, and MP powder, with an advantage to OP yogurt in all sensorial properties. At a level of 5%, deterioration in flavor, color, texture, shape, and overall acceptability was observed. The overall acceptability of OP-yogurt (1%) had a score of 9.7, which decreased with gradient additions and the storage period, reaching 8.2 in OP-yogurt (5%) at the end of storage. Other yogurt samples come afterward.

**Table 6:** Sensory evaluation of yogurt supplemented with lemon, mandarin, orange pomaces powder (1, 3, and 5%) during cold preservation (mean ± SD).

	**Samples**	**Storage (days)**	**Flavor**	**Color**	**Texture**	**Shape**	**Over acceptability**
	Control	0	9.5 ± 0.1a	9.6 ± 0.3a	9.4 ± 0.5a	9.3 ± 0.3a	9.5 ± 0.2a
		10	9.1 ± 0.3b	9.2 ± 0.2b	9.0 ± 0.5b	9.0 ± 0.4b	9.1 ± 0.1b
		21	8.6 ± 0.2c	8.7 ± 0.1c	8.5 ± 0.3c	8.5 ± 0.2c	8.6 ± 0.3c
LP	Y1%	0	9.5 ± 0.0ab	9.6 ± 0.2a	9.5 ± 0.4a	9.5 ± 0.5a	9.5 ± 0.4a
		10	9.2 ± 0.3b	9.3 ± 0.2b	9.1 ± 0.6b	9.1 ± 0.3b	9.2 ± 0.3b
		21	8.8 ± 0.1c	8.7 ± 0.0c	8.6 ± 0.7c	8.4 ± 0.2cd	8.6 ± 0.2c
	Y3%	0	9.6 ± 0.2a	9.7 ± 0.0a	9.4 ± 0.3a	9.3 ± 0.2ab	9.5 ± 0.4a
		10	8.9 ± 0.3c	9.2 ± 0.2b	9.0 ± 0.4b	8.8 ± 0.3c	9.0 ± 0.3b
		21	8.2 ± 0.5d	8.4 ± 0.1d	8.5 ± 0.6c	8.0 ± 0.2d	8.3 ± 0.2d
	Y5%	0	9.0 ± 0.0b	9.0 ± 0.2b	9.1 ± 0.1b	9.2 ± 0.0b	9.2 ± 0.3b
		10	8.5 ± 0.3cd	8.6 ± 0.3c	8.4 ± 0.2c	8.1 ± 0.2d	8.6 ± 0.4c
		21	8.1 ± 0.4d	8.2 ± 0.6d	7.9 ± 0.3d	7.7 ± 0.6e	8.0 ± 0.2d
MP	Y1%	0	9.6 ± 0.01a	9.7 ± 0.1a	9.5 ± 0.2a	9.6 ± 0.1a	9.6 ± 0.2a
		10	9.1 ± 0.2b	9.3 ± 0.3b	9.2 ± 0.01b	9.2 ± 0.2b	9.2 ± 0.1bc
		21	8.9 ± 0.1c	9.0 ± 0.2bc	8.8 ± 0.3c	8.9 ± 0.1c	8.9 ± 0.2c
	Y3%	0	9.4 ± 0.01ab	9.5 ± 0.4ab	9.3 ± 0.0b	9.3 ± 0.2b	9.4 ± 0.1b
		10	9.0 ± 0.2b	9.0 ± 0.6bc	8.6 ± 0.1c	9.0 ± 0.4bc	8.9 ± 0.3c
		21	8.3 ± 0.5d	8.5 ± 0.7c	8.1 ± 0.3d	8.7 ± 0.3c	8.4 ± 0.1d
	Y5%	0	9.1 ± 0.2b	9.0 ± 0.2bc	9.3 ± 0.5b	9.2 ± 0.3b	9.2 ± 0.2bc
		10	8.7 ± 0.3c	8.5 ± 0.2c	8.6 ± 0.7c	8.4 ± 0.4d	8.6 ± 0.4cd
		21	8.2 ± 0.2d	8.3 ± 0.3d	8.0 ± 0.2d	7.9 ± 0.6d	8.1 ± 0.1d
OP	Y1%	0	9.7 ± 0.0a	9.7 ± 0.2a	9.8 ± 0.03a	9.6 ± 0.2a	9.7 ± 0.0a
		10	9.2 ± 0.2b	9.3 ± 0.4b	9.4 ± 0.2b	9.2 ± 0.2b	9.3 ± 0.1b
		21	8.9 ± 0.4c	8.9 ± 0.3c	9.0 ± 0.03bc	8.8 ± 0.3c	8.9 ± 0.1c
	Y3%	0	9.3 ± 0.5b	9.5 ± 0.3ab	9.4 ± 0.02b	9.2 ± 0.1b	9.4 ± 0.2b
		10	9.0 ± 0.2b	9.1 ± 0.1bc	9.2 ± 0.09bc	8.8 ± 0.2c	9.0 ± 0.4c
		21	8.7 ± 0.1c	8.9 ± 0.2c	8.8 ± 0.1c	8.2 ± 0.5d	8.7 ± 0.3cd
	Y5%	0	9.2 ± 0.3b	9.3 ± 0.3b	9.1 ± 0.3bc	9.0 ± 0.5b	9.2 ± 0.4bc
		10	8.7 ± 0.5c	8.9 ± 0.6c	8.5 ± 0.2c	8.3 ± 0.4d	8.6 ± 0.5cd
		21	8.3 ± 0.4d	8.4 ± 0.0d	8.2 ± 0.3d	8.0 ± 0.1d	8.2 ± 0.3d

Means with different lowercase in the same column indicate significant differences between sensorial quality of yogurt samples p < 0.05.

**Figure 4:**
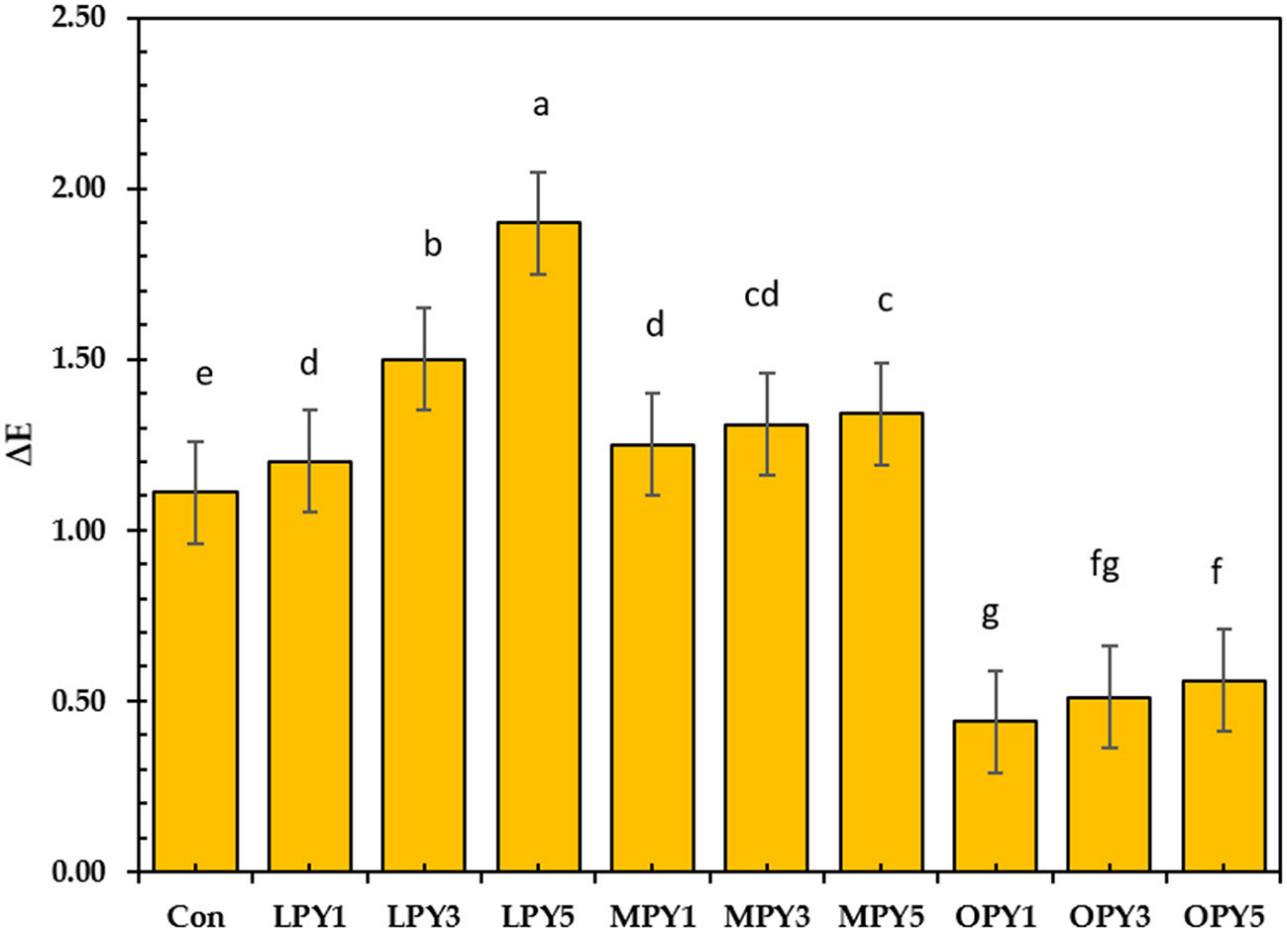
Color changes (ΔE) of yogurt supplemented with lemon, mandarin, orange pomaces powder (1, 3, and 5%) during cold preservation. Data presented mean ±SD, Lowercase letters above column indicate signficant differences.

Figure 4 shows the changes in the color of yogurt supplemented with different concentrations of OP, LP, and MP powder during cold storage periods at (4°C) after 21 days. OP-yogurt considerably maintained the yogurt color compared to control, while LP-yogurt showed higher values of color change compared to other samples. At the beginning of storage, the sensory scores of all yogurt samples were high due to their more intense flavors and better consistency. However, after 14 days, the acidity of the yogurt samples increased, and the sensory scores of all yogurt samples began to decrease. The overall acceptability scores of yogurts increased during storage for up to 14 days and then decreased. Thus, it may be attributed to the development of acidity. In general, yogurt supplemented with different levels of OP, LP, and MP powder had acceptable results during storage. These results can be applied to the development of functional yogurt with excellent antioxidant, antimicrobial, and anti-tumor activities without affecting the sensory characteristics of fermented milk, improving the final product's flavor characteristics and masking the defects through the natural development of aroma. Salehi (60) found that citrus fiber-enriched fermented milk has good acceptability and is an excellent vehicle for several commercial probiotics.

According to Gahruie et al. (62), despite the fiber-related health benefits, it is precious to note that consumers do not usually accept formulations with more than 3% fiber. From these results, it could be revealed that OP, LP, and MP powder could be added to fermented milk at levels of 1% and 3% to obtain products having the best scores for all the evaluated. According to Ivanova et al. (63), the gradient addition of pectin from 0.1 to 0.3% altered sensory perception, but high sensory scores were demonstrated in yogurt samples supplemented with 0.20% celery pectin.

#### Microbial changes during cold storage of yogurt samples

**Figure 5:**
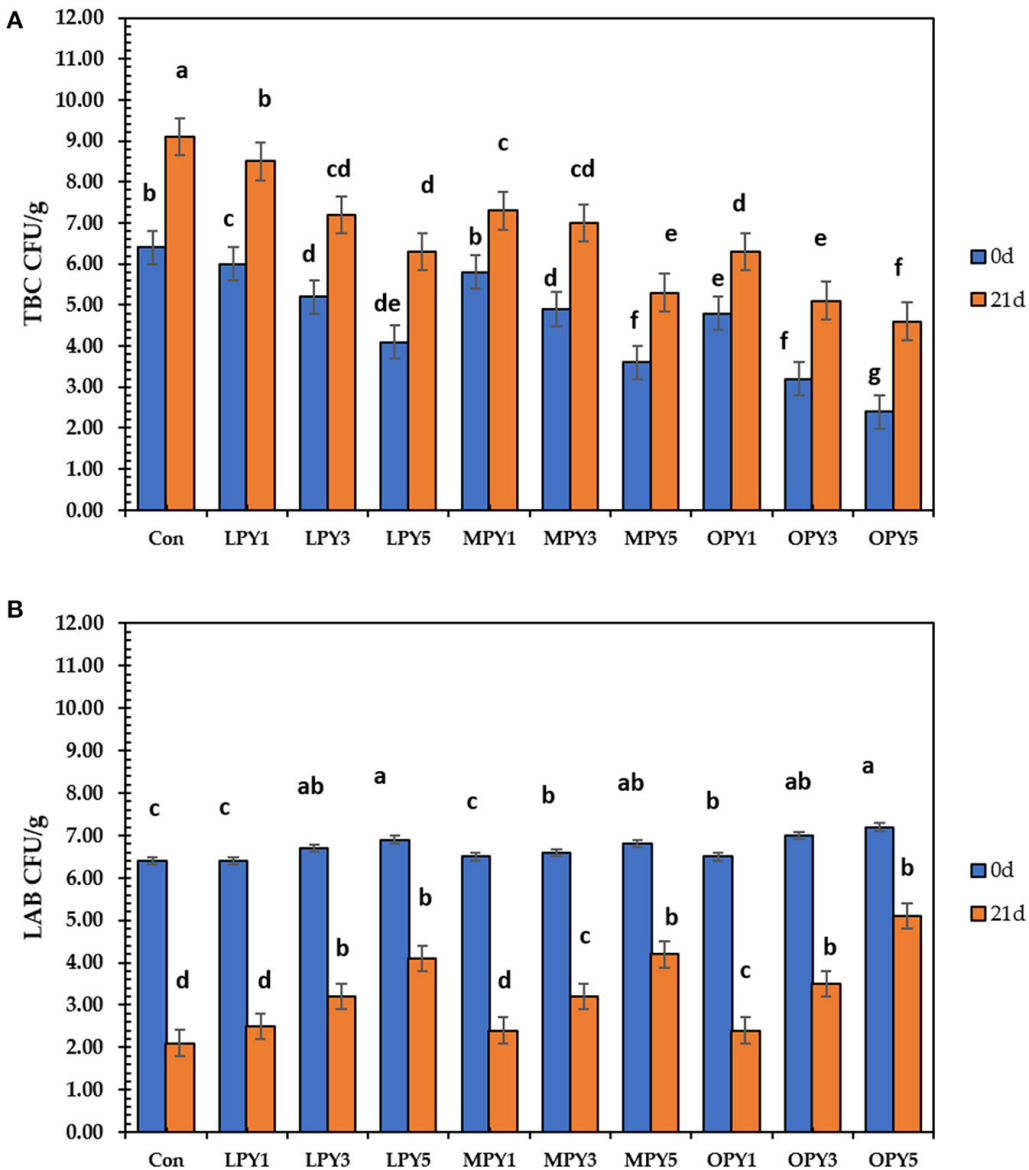
Total bacterial **(A)** and lactic acid bacterial count **(B)** in yogurt supplemented with lemon, mandarin, orange pomaces powder (1, 3, and 5%) during cold preservation. Data presented mean ±SD, black lowercase letters above column indicate signficant differences at 0 and 21 days of storage.

Results in Figure 5 showed that all samples had adequate amounts of viable lactic acid bacteria until 21 days of storage. So, all yogurt samples are satisfactory for 3 weeks. The lactic acid bacteria (LAB) count was decreased during storage. After 21-day storage, the LAB count decreased by 67% in control, but in OPY, 5% increased LAB count by 58% compared to control, which increased depending on citrus pomace level. The presence of probiotic bacteria (*Bifidobacterium* Bb12*, Lactobacillus acidophilus* LA-5, and *Streptococcus thermophilus*) in yogurt after 21 days of cold storage at 4°C may be linked to the low pH post-acidification. The yogurt starter lives in mutual stimulation related to aromatic compounds' growth, acidification, and production. After fermentation, organic acid accumulation (e.g., lactic and acetic acid) occurs.

Hu et al. (64) reported that organic acids are dominant antimicrobial agents. The microbial profile of citrus pomace-supplemented yogurt at 21 days of cold storage, Yeast, molds, coliform group, proteolytic and lipolytic bacteria in fermented yogurt samples have been detected, ensuring the hygienic-sanitary safety of the yogurt. This study demonstrated that the inclusion of citrus pomace contributes to the growth of probiotics in yogurt samples and reduces harmful bacteria (Figure 5).

## Conclusion

The accumulation of organic waste adversely affects the environment and health. In this study, citrus pomace, which is a waste product from juice factories, was used to make a supplement with extra nutrients. The presence of natural phenolics, flavonoids, antioxidants, and functional dietary fiber. OP exerted several biological activities in this study compared to MP and LP. The addition of OP to yogurt formulation at 5% considerably enhanced LAB count by 58% compared to control, physiochemical properties, i.e., the fat content that enhances stability and texture of yogurt, also the sensorial quality was enhanced. Orange, mandarin, and lemon peel powder permit their application in food processing to get healthy products and are used as a natural antioxidant to increase the shelf life of food products. Using food waste to make yogurt is a good idea because it gives people more access to healthier foods. This study may reference yogurt manufacturers looking forward to mixing novel prebiotics with probiotics.

## Data availability statement

The raw data supporting the conclusions of this article will be made available by the authors, without undue reservation.

## Author contributions

SA, AS, NA, and GA: conceptualization. GA, DA-Q, NB, and MA: data curation. AS, NA, HA, and AB: formal analysis. MOA, HA, AB, MHA, MSA, HSG, and SS: methodology. DA-Q, NB, MA, MOA, HA, AB, and MHA: investigation. SA, HA, MSA, HSG, and SS: resources. SA and AS: supervision. AS: writing—original draft. SA, AS, and SS: writing—review and editing. All authors have read and agreed to the published version of the manuscript.

## Conflict of interest

The authors declare that the research was conducted in the absence of any commercial or financial relationships that could be construed as a potential conflict of interest.

## Publisher's note

All claims expressed in this article are solely those of the authors and do not necessarily represent those of their affiliated organizations, or those of the publisher, the editors and the reviewers. Any product that may be evaluated in this article, or claim that may be made by its manufacturer, is not guaranteed or endorsed by the publisher.

## References

[1] Barber TM Kabisch S Pfeiffer AF Weickert MO. The health benefits of dietary fibre. Nutrients. (2020) 12:3209. 10.3390/nu12103209 33096647 PMC7589116

[2] Rohm H Brennan C Turner C Günther E Campbell G Hernando I. Adding value to fruit processing waste: Innovative ways to incorporate fibers from berry pomace in baked and extruded cereal-based foods—a susfood project. Foods. (2015) 4:690–7. 10.3390/foods4040690 28231231 PMC5224562

[3] Struck S Gundel L Zahn S Rohm H. Fiber enriched reduced sugar muffins made from iso-viscous batters. LWT. (2016) 65:32–8. 10.1016/j.lwt.2015.07.053

[4] Iqbal A Schulz P Rizvi SS. Valorization of bioactive compounds in fruit pomace from agro-fruit industries: Present Insights and future challenges. Food Biosci. (2021) 44:101384. 10.1016/j.fbio.2021.101384

[5] Poli A Anzelmo G Fiorentino G Nicolaus B Tommonaro G Di Donato P. Polysaccharides from wastes of vegetable industrial processing: new opportunities for their eco-friendly re-use. Biotechnol Biopolymers. (2011) 33:56. 10.5772/16387

[6] Khan UM Sameen A Aadil RM Shahid M Sezen S Zarrabi A. Citrus genus and its waste utilization: a review on health-promoting activities and industrial application. Evid Based Complement Altern Med. (2021) 2021:2488804. 10.1155/2021/2488804 34795782 PMC8595006

[7] Eliaz I Raz A. Pleiotropic effects of modified citrus pectin. Nutrients. (2019) 11:2619. 10.3390/nu11112619 31683865 PMC6893732

[8] Deng M Lin Y Dong L Jia X Shen Y Liu L. Physicochemical and functional properties of dietary fiber from pummelo (*Citrus Grandis* L. Osbeck) and grapefruit (Citrus Paradisi Mcfad) cultivars. Food Biosci. (2021) 40:100890. 10.1016/j.fbio.2021.100890

[9] Kirbaş Z Kumcuoglu S Tavman S. Effects of apple, orange and carrot pomace powders on gluten-free batter rheology and cake properties. J Food Sci Technol. (2019) 56:914–26. 10.1007/s13197-018-03554-z 30906049 PMC6400728

[10] Rezende ESV Lima GC Naves MMV. Dietary fibers as beneficial microbiota modulators: a proposed classification by prebiotic categories. Nutrition. (2021) 89:111217. 10.1016/j.nut.2021.111217 33838493

[11] Suri S Singh A Nema PK. Current applications of citrus fruit processing waste: a scientific outlook. Appli Food Res. (2022) 2:100050. 10.1016/j.afres.2022.100050

[12] Yun D Liu J. Recent Advances on the development of food packaging films based on citrus processing wastes: a review. J Agric Food Res. (2022) 9:100316. 10.1016/j.jafr.2022.100316

[13] Ashaolu TJ. Immune boosting functional foods and their mechanisms: a critical evaluation of probiotics and prebiotics. Biomed Pharmacother. (2020) 130:110625. 10.1016/j.biopha.2020.110625 32795926

[14] Mohammadi R Mortazavian AM. Technological aspects of prebiotics in probiotic fermented milks. Food Rev Int. (2011) 27:192–212. 10.1080/87559129.2010.535235

[15] Fontana L Bermudez-Brito M Plaza-Diaz J Munoz-Quezada S Gil A. Sources, isolation, characterisation and evaluation of probiotics. Br J Nutr. (2013) 109:S35–50. 10.1017/S0007114512004011 23360880

[16] El-Saadony MT Sitohy MZ Ramadan MF Saad AM. Green nanotechnology for preserving and enriching yogurt with biologically available iron (Ii). Innov Food Sci Emerg Technol. (2021) 69:102645. 10.1016/j.ifset.2021.102645

[17] Singh B Singh JP Kaur A Singh N. Phenolic composition, antioxidant potential and health benefits of citrus peel. Food Res Int. (2020) 132:109114. 10.1016/j.foodres.2020.109114 32331689

[18] Namir M Iskander A Alyamani A Sayed-Ahmed ETA Saad AM Elsahy K. Upgrading common wheat pasta by fiber-rich fraction of potato peel byproduct at different particle sizes: effects on physicochemical, thermal, and sensory properties. Molecules. (2022) 27:2868. 10.3390/molecules27092868 35566217 PMC9101751

[19] Saad AM El-Saadony MT Mohamed AS Ahmed AI Sitohy MZ. Impact of cucumber pomace fortification on the nutritional, sensorial and technological quality of soft wheat flour-based noodles. Int J Food Sci Technol. (2021) 56:3255–68. 10.1111/ijfs.14970

[20] Yao J Chen J Yang J Hao Y Fan Y Wang C. Free, soluble-bound and insoluble-bound phenolics and their bioactivity in raspberry pomace. LWT. (2021) 135:109995. 10.1016/j.lwt.2020.109995

[21] AOAC. Official Methods of Analysis of Aoac International. 19th Edn. Washington, DC: Aoac. (2012).

[22] McCleary BV DeVries JW Rader JI Cohen G Prosky L Mugford DC. Determination of insoluble, soluble, and total dietary fiber (Codex Definition) by enzymatic-gravimetric method and liquid chromatography: collaborative study. J AOAC Int. (2012) 95:824–44. 10.5740/jaoacint.CS2011_25 22816275

[23] Namir M Siliha H Ramadan MF. Fiber pectin from tomato pomace: characteristics, functional properties and application in low-fat beef burger. J Food Meas Charact. (2015) 9:305–12. 10.1007/s11694-015-9236-5

[24] Saad AM El-Saadony MT El-Tahan AM Sayed S Moustafa MA Taha AE. Polyphenolic extracts from pomegranate and watermelon wastes as substrate to fabricate sustainable silver nanoparticles with larvicidal effect against *Spodoptera littoralis*. Saudi J Biol Sci. (2021) 28:5674–83. 10.1016/j.sjbs.2021.06.011 34588879 PMC8459111

[25] Hassanin AA Saad AM Bardisi EA Salama A Sitohy MZ. Transfer of anthocyanin accumulating delila and rosea1 genes from the transgenic tomato micro-tom cultivar to moneymaker cultivar by conventional breeding. J Agric Food Chem. (2020) 68:10741–9. 10.1021/acs.jafc.0c03307 32833446

[26] Saad AM Mohamed AS El-Saadony MT Sitohy MZ. Palatable functional cucumber juices supplemented with polyphenols-rich herbal extracts. LWT. (2021) 148:111668. 10.1016/j.lwt.2021.111668

[27] El-Saadony MT Saad AM Elakkad HA El-Tahan AM Alshahrani OA Alshilawi MS. Flavoring and extending the shelf life of cucumber juice with aroma compounds-rich herbal extracts at 4°C through controlling chemical and microbial fluctuations. Saudi J Biol Sci. (2022) 29:346–54. 10.1016/j.sjbs.2021.08.092 35002428 PMC8717152

[28] El-Saadony MT Osama SF Khalil Ali Osman Alshilawi MS Taha AE Aboelenin SM. Bioactive peptides supplemented raw buffalo milk: biological activity, shelf life and quality properties during cold preservation Saudi. J Biol Sci. (2021) 28:4581–91. 10.1016/j.sjbs.2021.04.065 34354444 PMC8325055

[29] Celik OF Con AH Saygin H Sahin N Temiz H. Solation and identification of lactobacilli from traditional yogurts as potential starter cultures. LWT. (2021) 148:111774. 10.1016/j.lwt.2021.111774

[30] Gu Y Xing Li Chen H Guan K Qi X Yang L. Evaluation of faas and ffas in yogurts fermented with different starter cultures during storage. J Food Compos Anal. (2021) 96:103666. 10.1016/j.jfca.2020.103666

[31] Chau CF Huang YL. Comparison of the chemical composition and physicochemical properties of different fibers prepared from the peel of *Citrus Sinensis* L. Cv Liucheng. J Agric Food Chem. (2003) 51:2615–8. 10.1021/jf025919b 12696946

[32] Antonic B Jancikova S Dordevic D Tremlova B. Apple pomace as food fortification ingredient: a systematic review and meta-analysis. J Food Sci Technol. (2020) 85:2977–85. 10.1111/1750-3841.15449 32966605

[33] Wang X Kristo E LaPointe G. The effect of apple pomace on the texture, rheology and microstructure of set type yogurt. Food Hydrocoll. (2019) 91:83–91. 10.1016/j.foodhyd.2019.01.004

[34] Bchir B Rabetafika HN Paquot M Blecker C. Effect of pear, apple and date fibres from cooked fruit by-products on dough performance and bread quality. J Food Bioproc Tech. (2014) 7:1114–27. 10.1007/s11947-013-1148-y

[35] Dey D Richter JK Ek P Gu BJ Ganjyal GM. Utilization of food processing by-products in extrusion processing: a Review. Front Sustain Food Syst. (2021) 4:603751. 10.3389/fsufs.2020.60375129787291

[36] Dong X Hu Y Li Y Zhou Z. The maturity degree, phenolic compounds and antioxidant activity of eureka lemon [*Citrus Limon* (L.) Burm. F.]: A negative correlation between total phenolic content, antioxidant capacity and soluble solid content. Sci Hortic. (2019) 243:281–9. 10.1016/j.scienta.2018.08.036

[37] Zeb A. Concept, mechanism, and applications of phenolic antioxidants in foods. J Food Biochem. (2020) 44:e13394. 10.1111/jfbc.13394 32691460

[38] Wang L Lee WW Yang HW Ryu BM Cui YR Lee SC. Protective effect of water extract of citrus pomace against aaph-induced oxidative stress *in vitro* in vero cells and *in vivo* in zebrafish. J Prev Nutr Food Sci. (2018) 23:301–8. 10.3746/pnf.2018.23.4.301 30675459 PMC6342543

[39] Wang L Jo MJ Katagiri R Harata K Ohta M Ogawa A. Antioxidant effects of citrus pomace extracts processed by super-heated steam. LWT. (2018) 90:331–8. 10.1016/j.lwt.2017.12.024

[40] Fernández-Fernández AM Dellacassa E Nardin T Larcher R Gámbaro A Medrano-Fernandez A. *In vitro* bioaccessibility of bioactive compounds from citrus pomaces and orange pomace biscuits. Molecules. (2021) 26:3480. 10.3390/molecules26123480 34201056 PMC8229244

[41] Sir Elkhatim KA Elagib RA Hassan AB. Content of phenolic compounds and vitamin c and antioxidant activity in wasted parts of sudanese citrus fruits. Food Sci Nutr. (2018) 6:1214–9. 10.1002/fsn3.660 30065822 PMC6060895

[42] Nayak B Dahmoune F Moussi K Remini H Dairi S Aoun O. Comparison of microwave, ultrasound and accelerated-assisted solvent extraction for recovery of polyphenols from *Citrus Sinensis* Peels. Food Chem. (2015) 187:507–16. 10.1016/j.foodchem.2015.04.081 25977057

[43] Das AK Nanda PK Chowdhury NR Dandapat P Gagaoua M Chauhan P. Application of pomegranate by-products in muscle foods: oxidative indices, colour stability, shelf life and health benefits. Molecules. (2021) 26:467. 10.3390/molecules26020467 33477314 PMC7830841

[44] Chen Y Pan H Hao S Pan D Wang G Yu W. Evaluation of phenolic composition and antioxidant properties of different varieties of chinese citrus. Food Chem. (2021) 364:130413. 10.1016/j.foodchem.2021.130413 34175629

[45] Montenegro-Landívar MF Tapia-Quirós P Vecino X Reig M Valderrama C Granados M. Recovery of added-value compounds from orange and spinach processing residues: green extraction of phenolic compounds and evaluation of antioxidant activity. Antioxidants. (2021) 10:1800. 10.3390/antiox10111800 34829670 PMC8614849

[46] Sharma K Mahato N Cho MH Lee YR. Converting citrus wastes into value-added products: economic and environmently friendly approaches. Nutrition. (2017) 34:29–46. 10.1016/j.nut.2016.09.006 28063510

[47] Espina L Somolinos M Lorán S Conchello P García D Pagán R. Chemical composition of commercial citrus fruit essential oils and evaluation of their antimicrobial activity acting alone or in combined processes. Food Control. (2011) 22:896–902. 10.1016/j.foodcont.2010.11.021

[48] Panzella L Moccia F Nasti R Marzorati S Verotta L Napolitano A. Bioactive phenolic compounds from agri-food wastes: an update on green and sustainable extraction methodologies. Front Nutr. (2020) 7:60. 10.3389/fnut.2020.00060 32457916 PMC7221145

[49] Ebbensgaard A Mordhorst H Aarestrup FM Hansen EB. The role of outer membrane proteins and lipopolysaccharides for the sensitivity of *Escherichia coli* to antimicrobial peptides. Front Microbiol. (2018) 9:2153. 10.3389/fmicb.2018.02153 30245684 PMC6137088

[50] Min KY Kim HJ Lee KA Kim KT Paik HD. Antimicrobial activity of acid-hydrolyzed *Citrus unshiu* peel extract in milk. J Dairy Sci. (2014) 97:1955–60. 10.3168/jds.2013-7390 24534507

[51] Caputo L Quintieri L Cavalluzzi MM Lentini G Habtemariam S. Antimicrobial and antibiofilm activities of citrus water-extracts obtained by microwave-assisted and conventional methods. Biomedicines. (2018) 6:70. 10.3390/biomedicines6020070 29914193 PMC6026940

[52] Casas Cardoso L Cejudo Bastante C Mantell Serrano C Martínez de la Ossa EJ. Application of citrus by-products in the production of active food packaging. Antioxidants. (2022) 11:738. 10.3390/antiox11040738 35453422 PMC9028817

[53] Oikeh EI Oviasogie FE Omoregie ES. Quantitative phytochemical analysis and antimicrobial activities of fresh and dry ethanol extracts of Citrus sinensis (L) Osbeck (sweet orange) peels. Clin Phytoscience. (2020) 6:1–6. 10.1186/s40816-020-00193-w

[54] Fiorentini C Duserm Garrido G Bassani A Cortimiglia C Zaccone M Montalbano L. Citrus peel extracts for industrial-scale production of bio-based active food packaging. Foods. (2021) 11:30. 10.3390/foods11010030 35010155 PMC8750968

[55] Jan R. Understanding apoptosis and apoptotic pathways targeted cancer therapeutics. Adv Pharm Bull. (2019) 9:205–18. 10.15171/apb.2019.024 31380246 PMC6664112

[56] Tajaldini M Samadi F Khosravi A Ghasemnejad A Asadi J. Protective and anticancer effects of orange peel extract and naringin in doxorubicin treated esophageal cancer stem cell xenograft tumor mouse model. Biomed Pharmacother. (2020) 121:109594. 10.1016/j.biopha.2019.109594 31707344

[57] Nasser SA. The Addition of lemon peel powder affects the properties of yogurt. J Food Dairy Sci. (2022) 13:65–70. 10.21608/jfds.2022.136776.1055

[58] Khatoon N Ali S Liu N Muzammil HS. Preparation and quality assessment of fruit yoghurt with persimmon (Diospyros kaki): quality assessment of fruit yoghurt with persimmon. Proceed Pakistan Acad Sci: B. Life Environ Sci. (2021) 58:111–28. 10.53560/PPASB(58-1)583

[59] Arioui F Ait Saada D Cheriguene A. Physicochemical and sensory quality of yogurt incorporated with pectin from peel of *Citrus Sinensis*. Food Sci Nutr. (2017) 5:358–64. 10.1002/fsn3.400 28265371 PMC5332253

[60] Salehi F. Quality, physicochemical, and textural properties of dairy products containing fruits and vegetables: a review. Food Sci Nutr. (2021) 9:4666–86. 10.1002/fsn3.2430 34401112 PMC8358338

[61] Dello Staffolo M Sato AC Cunha RL. Utilization of plant dietary fibers to reinforce low-calorie dairy dessert structure. Food Bioproc Tech. (2017) 10:914–25. 10.1007/s11947-017-1872-9

[62] Gahruie HH Eskandari MH Mesbahi G Hanifpour MA. Scientific and technical aspects of yogurt fortification: a review. Food Sci Hum Wellness. (2015) 4:1–8. 10.1016/j.fshw.2015.03.002

[63] Nabiyeva Z Zhexenbay N Iskakova G Kizatova M Akhmetsadykova S. Development of dairy products technology with application low-etherificated pectin products. Eastern-European J Enter Technol. (2021) 3:111. 10.15587/1729-4061.2021.23382135592693

[64] Hu CH Ren LQ Zhou Y Ye BC. Characterization of antimicrobial activity of three *Lactobacillus Plantarum* strains isolated from chinese traditional dairy food. Food Sci Nutr. (2019) 7:1997–2005. 10.1002/fsn3.1025 31289647 PMC6593389

